# Usability of abattoir-acquired pig eyes for refractive excimer laser research

**DOI:** 10.1038/s41598-021-98635-z

**Published:** 2021-09-27

**Authors:** Marius Topka, Yao Zhang, Antonia Bock, Peter Riedel, Johannes Lörner, Alexander Hammer, Eva Maier, Friedrich Paulsen, Christian M. Hammer

**Affiliations:** 1 Institute of Functional and Clinical Anatomy, Friedrich-Alexander University Erlangen-Nürnberg, Universitätsstraße 19, 91054 Erlangen, Bavaria Germany; 2 WaveLight GmbH, Erlangen, Bavaria Germany; 3 Department of Neurosurgery, Paracelsus Medical University, Nürnberg, Bavaria Germany; 4 Dental Clinic 1, Operative Dentistry and Periodontology, Friedrich-Alexander University Erlangen-Nürnberg, Erlangen, Bavaria Germany; 5 Department of Operative Surgery and Topographic Anatomy, Sechenov University, Moscow, Russia

**Keywords:** Anatomy, Medical research

## Abstract

The purpose of this study was to elucidate, under which conditions abattoir-acquired pig eyes are suitable for refractive excimer laser experiments. Porcine eyes from tunnel-scalded (n = 5) and tank-scalded (n = 10) pigs were compared to unscalded eyes (n = 5) and to eyes scalded in the laboratory (n = 5). The corneal epithelium was removed before an excimer laser was used to perform a − 8.0 D photoablation. Corneal thickness was measured by optical coherence topography before and after photoablation. The ablation depth was determined with a contour measuring station, the morphology of the ablated areas was characterized by scanning electron microscopy and white-light profilometry. The scalded eyes showed an increase in corneal swelling which gained statistical significance in tank-scalded eyes showing a wedge-shaped opaque stromal lesion in the nasal corneal quadrant. A measurable deterioration of photoablation was only found in tank-scalded eyes that exhibited the opaque lesion. Ablated area morphology was smooth and regular in the unscalded and tunnel-scalded eyes. The tank-scalded eyes showed conspicuous wrinkles. While unscalded eyes should always be preferred for excimer laser laboratory experiments, the data suggest that the use of tunnel-scalded eyes may also be acceptable and should be chosen over tank-scalded eyes.

## Introduction

Pig eyes are frequently used as model systems for ophthalmological research. They share considerable similarities in shape and size with human eyes^1–5^. Usually, they are readily available from local slaughterhouses and can be obtained in large numbers and at low cost. This makes them also very attractive for preclinical ex vivo refractive laser testing and research. Therefore, porcine eyes have been used extensively for these purposes^6–9^. However, the quality of pig eyes obtained from different abattoirs may vary profoundly, depending on the post-mortal treatment of the carcasses until eye enucleation. Upon slaughter, pig carcasses are usually scalded for approximately 10 min with water heated to 60–70 °C. This is supposed to decimate superficial germs on the animals’ skin and to facilitate hair removal. Scalding is commonly achieved by immersion of the carcass in a scalding tank or by spraying the hanging carcass with hot water in a scalding tunnel. After the scalding process, the carcasses are usually scraped vigorously by engine-powered metal and rubber brushes to remove the animals’ hair. This step normally takes a few minutes and is also accompanied by spraying the animal with hot water. In 2005, Chinnery et al. have already reported band-like, horizontal lesions on the corneal surface of the majority of pig eyes they had obtained from their local slaughterhouse^10^. Upon enquiry, they learned that their porcine eyes had been enucleated after the scalding and scrubbing procedures. They conjectured that the described lesions were caused by these processes, with the horizontal orientation reflecting the palpebral fissure. They further deduced that the usage of such eyes in corneal research may be problematic. Upon request, some slaughterhouses in Germany are able to harvest “fresh” pig eyes prior to scalding (after the kill). This is very desirable if the eyes are supposed to be used for refractive laser research. However, in some locations, it is not possible to obtain unscalded pig eyes due to local regulations or for other practical reasons. This justifies concerns about the usability of porcine eyes taken from scalded animals for ex vivo refractive laser experiments. In the present study, the suitability of scalded pig eyes for refractive procedures involving corneal excimer laser ablation was investigated.

## Methods

### Study design

**Figure 1:**
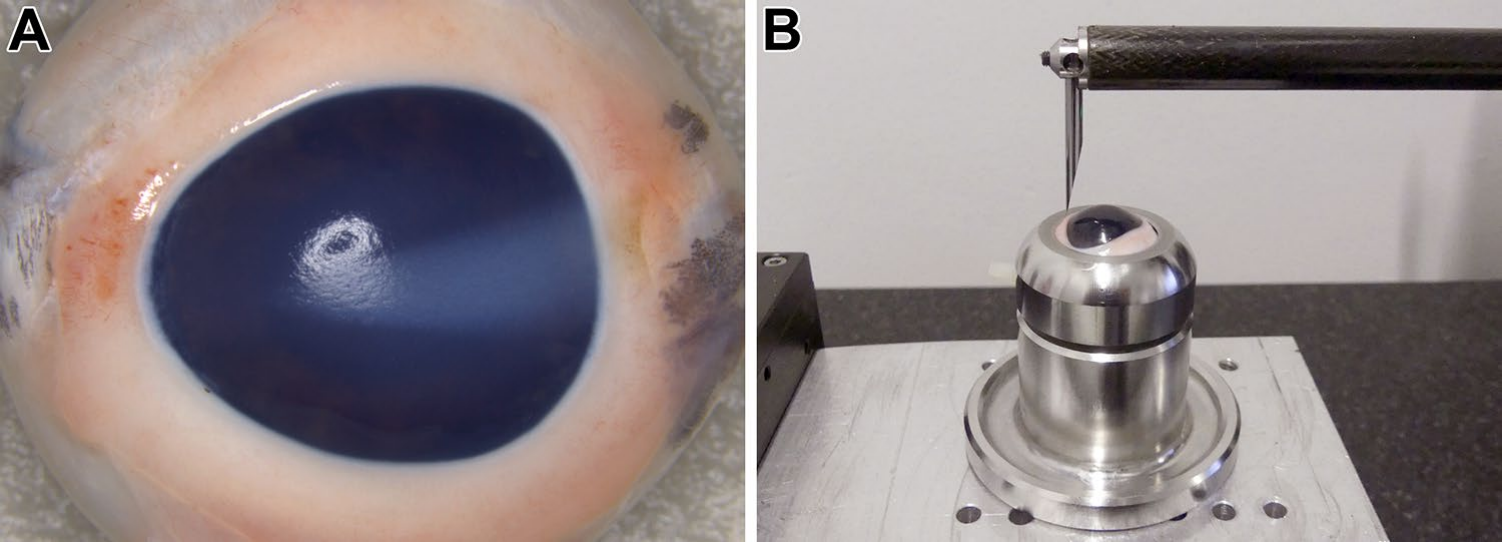
(**A**) Porcine eye with lesion taken from a tank-scalded pig. The wedge-shaped corneal opacity is clearly visible. (**B**) Probe arm of the MAHR contour measuring station over a porcine eye mounted on a custom-made holder.

A total of 25 porcine eyes taken from 25 animals (*Sus scrofa domestica*) aged approximately 0.5 years was subjected to excimer laser-assisted corneal ablation (− 8.0 D) after mechanical abrasion of the corneal epithelium. Of these, ten eyes had been harvested before the pigs being scalded. These “fresh” eyes were divided into a negative control group (group A, n = 5) and a positive control group (group D, n = 5). The negative control eyes remained unscalded until laser treatment and served as a reference. The positive control eyes were scalded for 10 min in the laboratory, immersed in one liter of phosphate buffered saline (PBS) heated to 65 °C. The remaining 15 eyes were taken from pigs that had been subjected to scalding and scraping at an abattoir approximately 5–10 min after slaughter (exsanguination). Five eyes were enucleated from pigs that had been scalded by spraying in a scalding tunnel (group B, n = 5). Ten eyes were harvested from pigs that had been scalded by immersion in a scalding tank (group C, n = 10). Notably, about 30–40% of the tank-scalded eyes showed a wedge-shaped and horizontally oriented opacity in the nasal quadrant of the cornea (Fig. 1A).

Therefore, group C was further subdivided into group C1 showing no opacity at all, and group C2 displaying the mentioned opacity. An overview of the experimental groups is given in Table 1.

**Table 1:** Overview of experimental groups (n = 5 each).

Group	Scalding	Description
Group A	No scalding	Negative control
Group B	Tunnel scalding followed by scraping	Scalded by spraying the carcass with hot water
Group C1	Tank scalding followed by scraping	Scalded by immersion of the carcass in hot water; without opaque corneal lesion
Group C2	Tank scalding followed by scraping	Scalded by immersion of the carcass in hot water; with opaque corneal lesion
Group D	Scalding in laboratory without subsequent scraping	Positive control

Prior to the experiments, the intraocular pressure was adjusted to 15 mm/Hg by intravitreal injection of PBS and controlled with an applanation tonometer (Tono-Pen XL; Medtronic, Jacksonville, FL, USA). After abrasion of the corneal epithelium, the corneal shape, central thickness, curvature, and structure were assessed before and after excimer laser ablation by anterior segment optical coherence tomography (AS-OCT) (TOMEY SS-1000, Tomey Inc., Nagoya, Japan) and by use of a mechanical contour measuring station (MarSurf XCR 20, MAHR GmbH, Göttingen, Germany). Moreover, the central ablation depth was determined with the contour measuring station. Finally, the eyes were immersed in a fixative containing 10% formalin and 2.5% glutaraldehyde for 24 h at room temperature and further processed for scanning electron microscopy (SEM). SEM was employed to morphologically analyze the surface of the ablated optical zone. After qualitative SEM analysis, quantitative data regarding the roughness of the ablation area (optical zone) were obtained applying 3D non-contact white-light profilometry on every specimen (Cyberscan CT100 with P-CHR-600 sensor (z = 0.02 µm), CyberTECHNOLOGIES, Eching, Germany). Comparison of the experimental groups described above was supposed to allow an evidence-based estimation on whether or not it is advisable to use tank- or tunnel-scalded eyes for laboratory experiments with refractive excimer lasers.

### Porcine eyes

The pig eyes used in this study were acquired from two different slaughterhouses. Unscalded and tunnel-scalded eyes were obtained on the same day from Contifleisch GmbH in Erlangen, Germany. Tank-scalded eyes were obtained from the municipal abattoir in Bamberg, Germany. The standard tunnel scalding procedure at the Erlangen abattoir encompassed spraying of the carcasses for 10–15 min with water heated to 60–62 °C. At the Bamberg slaughterhouse, the cadavers were immersed for 7 min in a tank filled with water heated to 60–62 °C. In both cases, the scalding process was ensued by scraping of the carcass with engine-powered metal and rubber brushes. During the scraping procedure, the carcasses were constantly sprayed with water heated to 60–70 °C. The duration of the scraping processes varied between 0.5 and 4 min, depending on how quickly the animal hairs were removed. Afterwards, the eyes were harvested by the abattoir staff and remained refrigerated (4 °C) until further use. The eyes of group D were obtained unscalded from the Erlangen abattoir. After removal of external eye muscles and fatty tissue, the eyes were scalded as a whole for 10 min, immersed in one liter of phosphate buffered saline (pH 7.35) heated to 65 °C. Subsequently, they were taken out of the hot PBS and allowed to cool down in a humid chamber until further use. Care was taken that all experiments were carried out within 6 h post-mortem. Prior to the experiments, all eyes were stored and transported in a humid chamber on ice to avoid desiccation of the samples.

### Excimer laser and contour measuring station parameters

The pig eyes were mounted on a custom-made holder as illustrated in Fig. 1. Holders and eyes were positioned on a contour measuring station (MarSurf XCR 20, MAHR GmbH, Göttingen, Germany) such that the vertically oriented tip of a probe arm (PCV 350 × 33 mm, MAHR GmbH, Göttingen, Germany) was moved centrally across the porcine cornea in a sagittal plane from inferior to superior by a drive unit (PCV 200, MAHR GmbH, Göttingen, Germany). The probe tip had a radius of 25 µm and exerted a constant compression force of 0.001 N on the corneal surface while being moved with a speed of 0.4 mm/s. To keep the compression force constant, the vertical z-position of the probe arm was constantly adjusted and recorded by the MAHR station. This way, the contour of the corneal surface was determined. The measured displacement of the probe arm was 12.0 mm, with spatial measurements taken in 1 µm intervals (12,000 measuring points for every contour line). Each porcine cornea was subjected to one contour measurement before and after excimer ablation. Comparison of the contour lines yielded an estimation of the shape of the ablated stroma. By comparison of the central z-positions of the pre- and post-ablation contour lines the central ablation depths were determined. Care was taken that the mounted eye was not moved at all between the two contour scans. To achieve this, the MAHR station was set up underneath the excimer laser which allowed for the two contour scans and the excimer ablation being performed without moving the mounted eye. Stromal ablation was carried out employing the WaveLight EX500® excimer laser platform (WaveLight GmbH, Erlangen, Germany). It was operated with a pulse energy of 1.75 mJ and a repetition rate of 500 Hz. The circular ablation zones had a diameter of 6.5 mm with a refractive correction aimed at − 8.0 D (central ablation depth aimed at 128.0 µm).

### Scanning electron microscopy

All eyes were immersion-fixed at room temperature for at least 24 h in PBS [pH 7.2] containing 10% formalin and 2.5% glutaraldehyde immediately after excimer ablation. The corneas were excised and trimmed for the region containing the ablation zone. The specimens were then subjected to post-fixation in 1% osmium tetroxide and dehydration in an ascending series of alcohols and acetone. Subsequently, the samples were critical point dried applying the Leica EM CPD300 system (Leica Mikrosysteme GmbH, Vienna, Austria). Then, the samples were mounted on aluminium stubs (Ted Pella Inc., Redding, CA, USA) by use of conductive silver (Plano GmbH, Wetzlar, Germany) and sputter coated with a 15 nm layer of gold using the Leica EM ACE200 system (Leica Mikrosysteme GmbH, Vienna, Austria). All specimens were analyzed with a JEOL scanning electron microscope (JSM-IT 300LV, JEOL Germany GmbH, Eching, Germany).

### White-light profilometry

After SEM analysis, the surface roughness of the optical zones was estimated quantitatively by application of the Cyberscan CT100 white-light profilometer (CyberTECHNOLOGIES, Eching, Germany) on all samples. For that purpose, a square area measuring 1 mm × 1 mm in the center of the optical zone was scanned with a measurement distance of 0.5 µm × 0.5 µm by use of the P-CHR-600 sensor (z = 0.02 µm). The mean roughness (*R*_*a*_) and mean roughness depth (*R*_*z*_) were determined for all specimens with the Scan Suite 8 software (CyberTECHNOLOGIES, Eching, Germany). Means and standard deviations of these values were calculated for each experimental group.

### Statistical analysis

Group-specific means and standard deviations were calculated for central corneal thickness, central ablation depth, mean roughness *R*_*a*_, and mean roughness depth *R*_*z*_. Differences between groups were tested for statistical significance with a *Bonferroni*-corrected univariate analysis of variance (one-way ANOVA) after confirmation of normal distribution of data with a *Kolmogorov–Smirnov* test and a *Shapiro–Wilk* test. p-values below 0.05 were considered statistically significant. For the statistical analyses performed in this study the SPSS statistics software (SPSS Statistics, version 26, International Business Machines Corp., Armonk, New York, USA) was used.

### Ethics declarations

No experiments in live animals were performed. Therefore, no approval by the local ethics committee for animal experiments was required. At the slaughterhouses, the pigs were treated according to the local municipal animal welfare regulations. This was supervised by the local departments of veterinary medicine and meat hygiene, which also sanctioned the acquisition of enucleated porcine eyes for research purposes.

## Results

### AS-OCT and contour measurements

**Figure 2:**
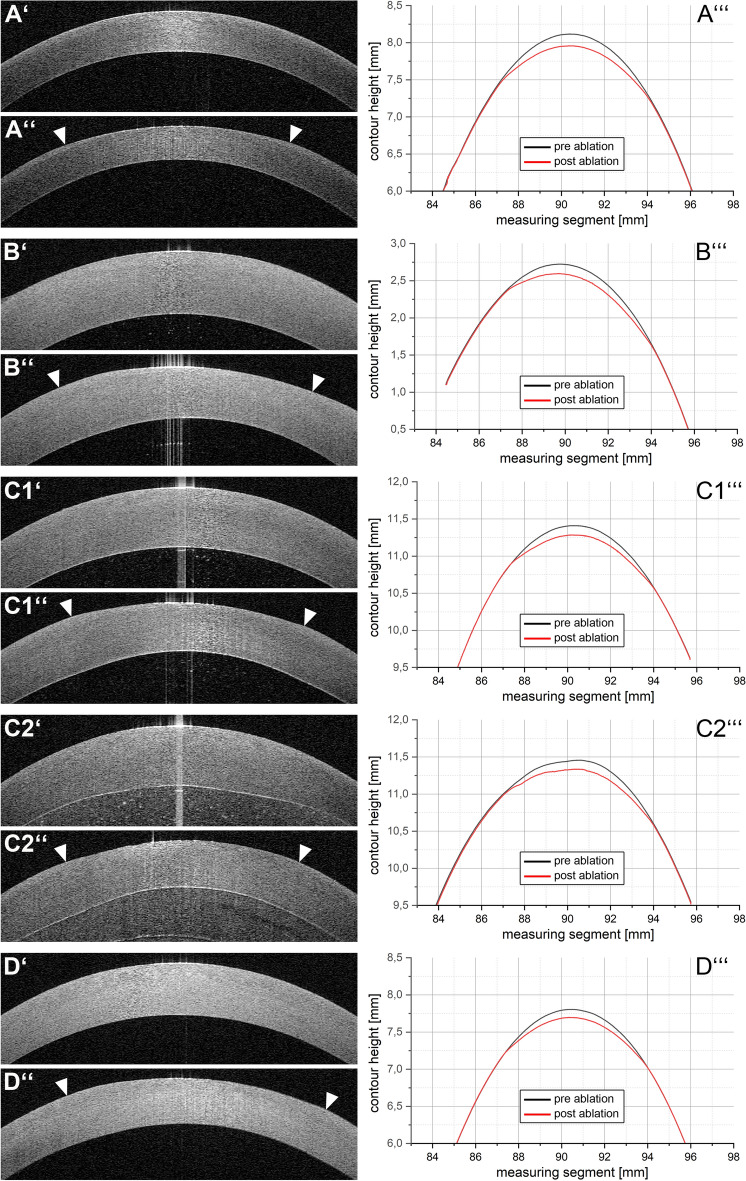
Representative AS-OCT images (left column) and contour diagrams (right column) of the porcine corneas before and after excimer ablation. (**A**) Group A (unscalded pigs); (**B**) group B (tunnel-scalded pigs); (**C1**) group C1 (tank-scalded pigs without corneal lesion); (**C2**) group C2 (tank-scalded pigs with corneal lesion); (**D**) group D (eyes scalded in the lab after enucleation). Pre-ablation AS-OCT images are indicated with one apostrophe, post-ablation AS-OCT images with two apostrophes. *Arrow heads* highlight the optical zone margin. Representative pre- and post-ablation MAHR contour measurements are shown in one diagram for every group and are indicated with three apostrophes.

Anterior segment optical coherence tomography (AS-OCT) revealed that excimer laser-driven stromal ablation was possible in all five experimental groups (Fig. 2). The pre-ablation AS-OCT images (Fig. 2A′–D′) display a regular, smooth, and evenly curved anterior surface of the porcine corneas. In all post-ablation AS-OCT images (Fig. 2A′′–D′′) the optical zone was discernible. In all scalded eyes, the corneal stroma exhibited a marked swelling when compared to the unscalded controls (group A). Comparative analysis of the central corneal thickness revealed that the swelling was most pronounced in group C2, followed by group C1, group B, and group D (see Table 2). The differences in mean central corneal thickness were statistically significant (p < 0.05) between group A and each of the other groups except group D. The mean central corneal thickness in group C2 was statistically different from the corresponding values in all other experimental groups (p < 0.05).

**Table 2:** Central corneal thickness in µm as obtained by AS-OCT before excimer laser ablation.

Eye-no.	Group A	Group B	Group C1	Group C2	Group D
1	762	1049	1092	1542	1041
2	830	925	1219	1533	975
3	818	995	1154	1400	904
4	778	1197	904	1149	1040
5	808	1190	1186	1257	1011
Mean	799.2^#^	1071.2*^#^	1111.0*^#^	1376.2*	994.2^#^
SD	28.3	120.0	124.8	172.1	57.2
Difference to A	0.0	272.0	311.8	577.0	195.0
Difference in %	0.0	25.4	28.1	41.9	19.6

The last two rows show the difference of the group-specific means in µm (second last row) and in per cent (last row).

*Statistically significant difference to Group A with p < 0.05 (Bonferroni-corrected one-way ANOVA).

^#^Statistically significant difference to Group C2 with p < 0.05 (Bonferroni-corrected one-way ANOVA).

Table 2 shows the central corneal thickness of every eye before laser treatment as well as the group-specific means (± standard deviation) for all experimental groups. It also shows the differences between the mean values of the unscalded group A and the scalded groups (B–D). Table 3 presents the central ablation depths as obtained by the contour measurement in every eye investigated. It also displays the group-specific means (± standard deviation) and shows that the central ablation depth was markedly reduced in groups C2 and D. However, only group D displayed a statistically significant difference in the mean central ablation when compared to group A (p = 0.027). With a value of p = 0.086, statistical significance was closely missed regarding the difference in mean central ablation depth between group A and group C2. The contours of the ablated corneas showed pronounced irregularities in group C2, as evident by the MAHR contour measurements (Fig. 2).

**Table 3:** Central ablation depth in µm as obtained by contour measurement.

Eye-no.	Group A	Group B	Group C1	Group C2	Group D
1	117.2	150.7	133.0	116.8	110.1
2	135.3	121.7	127.4	106.5	104.0
3	124.5	127.5	126.6	104.1	104.4
4	121.0	127.2	139.9	118.2	111.3
5	130.2	130.4	116.7	110.4	113.5
Mean	125.6^$^	131.5^#$^	128.7^#$^	111.2	108.7*
SD	7.2	11.2	8.6	6.2	4.3

*Statistically significant difference to Group A with p < 0.05 (Bonferroni-corrected one-way ANOVA).

^#^Statistically significant difference to Group C2 with p < 0.05 (Bonferroni-corrected one-way-ANOVA).

^$^Statistically significant difference to Group D with p < 0.05 (Bonferroni-corrected one-way ANOVA).

### Scanning electron microscopy

**Figure 3:**
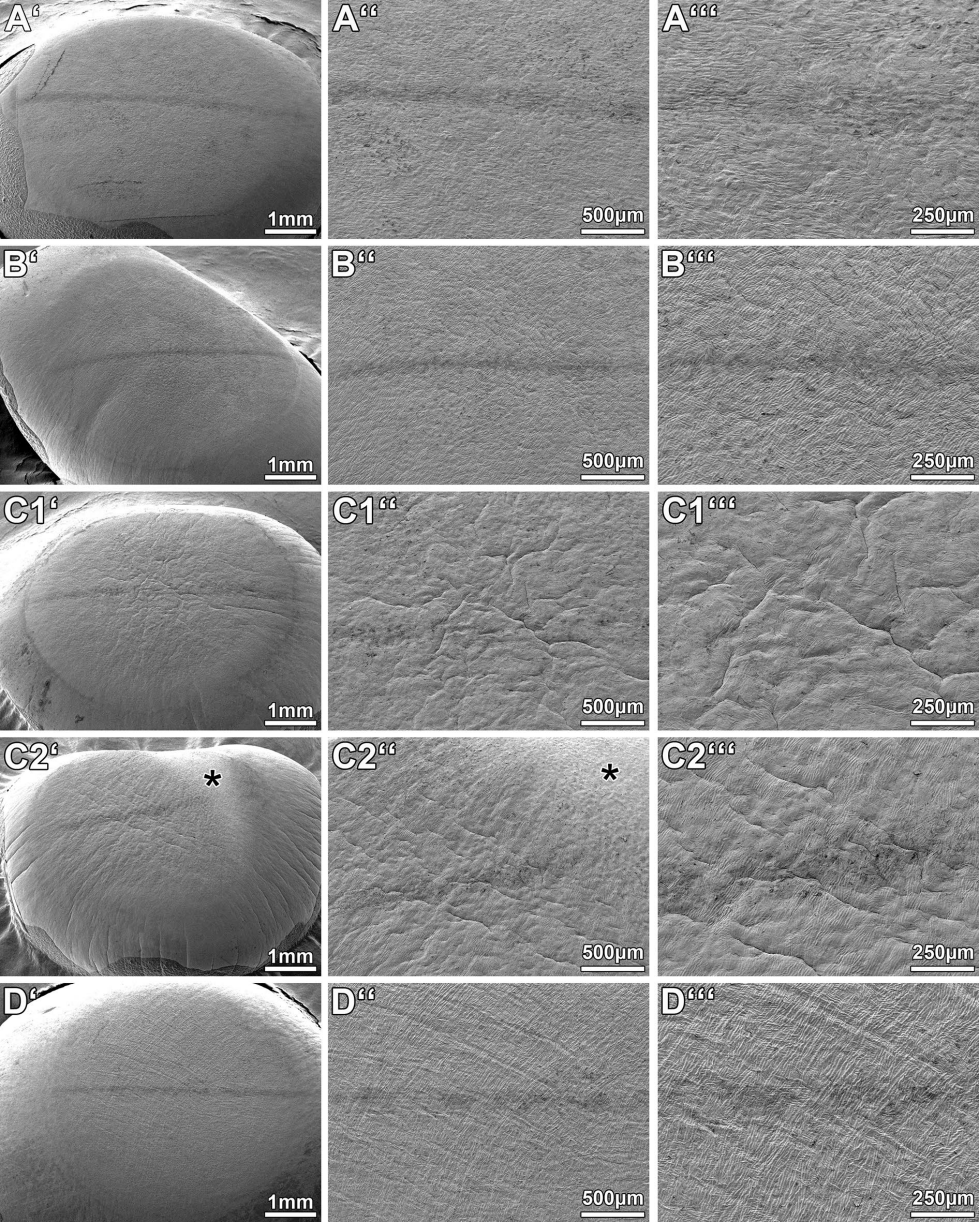
Representative scanning electron micrographs of porcine corneas after excimer ablation. (**A**) Group A (unscalded pigs); (**B**) group B (tunnel-scalded pigs); (**C1**) group C1 (tank-scalded pigs without corneal lesion); (**C2**) group C2 (tank-scalded pigs with corneal lesion); (**D**) group D (eyes scalded in the lab after enucleation). *Asterisk* corneal lesion in (**C2′,C2′′**). The path of the traversing probe of the MAHR contour measuring station is discernible as a dark, horizontal streak in all pictures.

The optical zone surfaces of the non-scalded samples (group A) and of the tunnel-scalded samples (group B) appeared smooth and regular (Fig. 3A,B). Occasional scrapes, like in Fig. 3A, most likely represent artifacts administered accidentally while handling the specimens. In the tank-scalded samples (groups C1 and C2) the optical zone surfaces showed minor wrinkles, but exhibited an otherwise reasonably smooth morphology (Fig. 3C1,C2). However, the wrinkles were not confined to the optical zone, but were also found in the sample periphery. The corneal lesions of group C2 were discernible by SEM as bright, wedge-shaped areas with a granular texture (Fig. 3C′,C′′). Most C2 specimens were markedly deformed by the critical point drying procedure (i.e. they buckled substantially). In group D the optical zones displayed conspicuous striae (Fig. 3D), but otherwise appeared similarly smooth as the specimens of group A and B. In all samples the path of the traversing MAHR probe was easily discernible as a stromal scratch mark.

### White-light profilometry

Table 4 presents the measured *R*_*a*_ and *R*_*z*_ values as well as the group-specific means and standard deviations. Only the differences between group A and group D were found to be statistically significant.

**Table 4:** Mean roughness (*R*_*a*_) and mean roughness depth (*R*_*z*_) values as determined by white-light interferometry.

Eye-no.	Group A		Group B		Group C1		Group C2		Group D	
	*R*_*a*_	*R*_*z*_	*R*_*a*_	*R*_*z*_	*R*_*a*_	*R*_*z*_	*R*_*a*_	*R*_*z*_	*R*_*a*_	*R*_*z*_
1	0.71	4.80	0.75	5.98	1.08	6.74	1.01	7.32	1.32	11.71
2	0.80	5.63	1.08	8.88	1.20	9.64	1.16	9.78	1.31	12.76
3	0.80	6.04	0.94	8.10	1.59	13.39	1.25	11.54	1.99	19.41
4	0.74	5.42	1.12	9.91	0.79	6.19	1.26	11.56	1.36	12.61
5	0.82	6.67	0.69	5.44	1.11	7.05	1.30	12.90	0.83	5.91
Mean	0.77	5.71	0.92	7.66	1.15	8.60	1.20	10.62	1.36*	12.48*
SD	0.05	0.70	0.19	1.90	0.29	3.00	0.12	2.15	0.41	4.79

*Statistically significant difference to Group A with p < 0.05 (Bonferroni-corrected one-way ANOVA).

## Discussion

Ex vivo porcine eyes have become an indispensable model system for refractive laser research. Due to their availability and similarity to human eyes, enucleated pig eyes are being used worldwide to investigate corneal tissue interactions of refractive laser platforms. With very few exceptions, porcine eyes are obtained from slaughterhouses and therefore underlie local veterinary regulations that sometimes do not allow for a delivery of eyes taken from unscalded animals. Hence, the question if scalded pig eyes are suitable for refractive laser research has far-reaching implications. The current study focusses on refractive excimer lasers that are commonly employed for procedures like laser in situ keratomileusis (LASIK) or photorefractive keratectomy (PRK)^11,12^.

### Corneal swelling

The scalding procedures have a marked impact on corneal swelling. A certain degree of postmortal swelling is normal for the corneal stroma as the barrier function of the corneal epithelium and the dehydrating function of the pumping endothelium are impaired in the degenerating cells^13^. However, while the mean central corneal thickness in the unscalded group (group A) was about 800 µm, all groups of eyes that had been taken from scalded animals showed significantly higher values above 1000 µm. The least pronounced swelling was found after tunnel scalding (group B), where the statistically significant (p = 0.011) mean difference to group A was 272 µm. With respect to tank scalding, the results were bipartite. In corneas without the described opaque corneal lesion (group C1), the mean stromal swelling was around 312 µm and thus largely comparable to the tunnel-scalded eyes of group B (group A vs. group C1: p = 0.003). However, corneas exhibiting the opaque lesion in the nasal quadrant (group C2) showed the most pronounced swelling with a mean difference to group A of 577 µm (p = 0.0000009) and a mean central corneal thickness of around 1376 µm. It is very likely that the increased stromal swelling in scalded eyes is attributable to lesions of the corneal epithelium and the concomitant loss of its barrier function. Chinnery et al. described a horizontal band of damaged corneal epithelium in the region of the palpebral fissure in scalded pigs^10^. Similar lesions were also detected in the scalded eyes used in the present study. Our data suggest that the lesions to the corneal epithelium are most pronounced in tank-scalded eyes displaying the described wedge-like opaque lesion in the nasal quadrant (group C2). It is conceivable that the wedge-like lesion itself is caused by hot water entering the slightly opened nasal portion of the eyelids. Therefore, the assumption of extraordinarily pronounced damage to the corneal epithelium (and maybe also to the corneal endothelium) leading to exceptionally marked stromal swelling in this group seems plausible. Following this reasoning, immersion scalding of isolated eyes in the laboratory should elicit even more pronounced corneal damage due to the total lack of the protective eyelids. Interestingly, the isolated eyes that had been scalded by immersion in hot buffer in the laboratory (group D) showed the smallest increase in central corneal thickness, which also did not reach statistical significance (p = 0.128). Perhaps this is an effect of thermally induced collagen denaturation, which has been demonstrated to harden connective tissue and even cause tissue shrinkage^14–17^. Both processes would limit or even counteract corneal swelling especially in the immersion scalded eyes, since here, the whole globes are surrounded by hot liquid. Therefore, the whole corneas and not only the regions of the palpebral fissure were exposed in this group.

### Central ablation depth

As the analysis of the central ablation depth suggests, excimer laser-driven photoablation is possible and yields satisfactory results in eyes taken from scalded pigs, irrespective of the scalding method. The mean ablation depth values within groups A, B, and C1 were close to the desired 128 µm (corresponding to − 8.0 D) and showed no statistically significant differences to each other. However, photoablation was impaired substantially in the tank-scalded eyes displaying the opaque nasal lesion (group C2) and after immersion scalding in the laboratory (group D). In both cases, the desired refractive outcome was missed by more than 1.0 D. Both, group C2 and group D, showed a statistically significant difference in mean central ablation depth when compared to groups B (tunnel scalding, C2: p = 0.006; D: p = 0.002) and C1 (tank scalding without nasal lesion, C2: p = 0.021; D: p = 0.006). When compared to the unscalded eyes of the negative control group A, the reduction in the mean central ablation depth was statistically significant in group D (p = 0.27), but not in group C2 (p = 0.86). It is very likely that the difference between group A and group C2 would have reached significance with a larger sample size. If the values of groups A, B, and C1 are pooled, the combined mean central ablation depth differs significantly from group C2 (p = 0.001).

### Optical zone surface morphology

Regarding the surface qualities of the ablated areas, the tunnel scalded eyes of group B showed the best results when compared to the other scalding groups. SEM analysis revealed very smooth and almost immaculate optical zones in the corneas taken from tunnel-scalded animals. Correspondingly, quantitative white light profilometry yielded only slightly increased mean *R*_*a*_ and *R*_*z*_ values that did not differ significantly from the negative control. As opposed to that, eyes taken from tank-scalded pigs showed conspicuous corneal wrinkles that may give reason for concern. However, these wrinkles were not confined to the optical zones but were observed all over the specimens, instead. This is indicative of the possibility that the wrinkles may represent artifacts caused by the tank-scalding process and/or by the preparation of the samples for SEM. A certain degree of tissue shrinkage normally occurs in samples undergoing fixation with formaldehyde and glutaraldehyde and/or the critical point drying procedure used for SEM^18^. However, a wise choice of fixation and drying parameters normally limits these phenomena. Perhaps, this is the reason, why no wrinkles were found in the unscalded and tunnel-scalded eyes. Therefore, it is very likely that the tank scalding procedure itself gave rise to the detected sample shrinkage. As of now, it is unknown whether the wrinkles were caused by tank scalding alone or by a combination with the fixation and drying procedures. Either way, the presence of corneal wrinkles may seriously limit the usability of eyes taken from tank-scalded animals for refractive excimer laser research. Interestingly, no such wrinkles were found after immersion scalding in the laboratory. It is tempting to speculate that the quick denaturation of the collagen in the entire outer shell of the globes prevented further shrinkage due to fixation and/or critical point drying in group D. Although the true nature of the small striae detected in the group D specimens remains unknown, it appears possible that they are a result of small-scale shrinkage. Obviously, these striae also manifest as substantially increased roughness parameters *R*_*a*_ and *R*_*z*_, as measured by white light profilometry.

### Limitations

Of course, the current study is limited by several factors. First and foremost, the limited sample size putatively prevented some of the obvious inter-group differences from reaching statistical significance. Nonetheless, the differences in ablation depth and optical zone morphology were considered convincing enough and worth reporting, especially since this is the first study to explore the effect of different scalding procedures on the excimer laser applicability. Secondly, the quantification of optical zone surface roughness was based on only one comparatively small region of interest (1 mm × 1 mm) in the center of the ablated area of each sample. This was owed to the fact that it is extremely difficult to obtain reliable and accurate roughness measurements with white light profilometry on a curved surface. Keeping the region of interest small reduced the confounding influence of the curvature. Other technologies like atomic force microscopy^19–23^ may allow for more accurate analyses of the corneal samples’ micro-topography. It could therefore represent a rewarding tool for the quantification of roughness parameters on curved surfaces in the future. Atomic force microscopy may also detect and quantify surface irregularities in the optical zones much better and gentler than the MAHR contour measuring station. However, due to the complexity of the technique, this remains a task to be accomplished by future research. Lastly, there remains an uncertainty of what really happened to the carcasses and to the porcine eyes in the abattoirs. Deviations from the standard scalding and scraping protocols would have remained unnoticed. Hence, possible influences of such deviations on the data obtained in the current study remained unaccounted for.

## Conclusion

The usage of fresh eyes taken from unscalded pigs is, of course, always preferable when it comes to excimer laser laboratory experiments on whole eyes. However, if this is not possible due to local circumstances, scalded eyes may serve as an adequate substitute, depending on the research question and on the quality of the eyes. The main findings of the present study suggest that tunnel scalding has a less detrimental effect on excimer laser photoablation than tank scalding. This conclusion is largely based on the excellent morphology of the optical zones and on the fact that tunnel-scalded eyes seem to be less prone to corneal swelling than tank scalded eyes. Nonetheless, if only tank scalded eyes are available, their application may also be tolerable under certain conditions. It is, however, imperative to screen the corneas of tank-scalded eyes for the described wedge-shaped opaque lesions in the nasal quadrant. As soon as these lesions are discernible, further utilization in excimer laser experiments is not recommended.

## Data Availability

The datasets generated during and/or analyzed during the current study are available from the corresponding author on reasonable request.
